# Activin A affects colorectal cancer progression and immunomodulation in a stage dependent manner

**DOI:** 10.1038/s41598-025-91853-9

**Published:** 2025-03-12

**Authors:** Mark B. Wiley, Jessica Bauer, Valentina Alvarez, Zoe Kolics, Wenxuan Cheng, David N. Church, David J. Kerr, Rachel S. Kerr, Barbara Jung

**Affiliations:** 1 Department of Medicine, University of Washington College of Medicine, Seattle, WA 98195 USA; 2 Nuffield Department of Medicine, University of Oxford, Oxford, OX1 4BH UK; 3 NIHR Oxford Comprehensive Biomedical Research Center, Oxford University Hospitals NHS Foundation Trust, University of Oxford, Oxford, OX1 4BH UK; 4 Radcliffe Department of Medicine, University of Oxford, Oxford, OX1 4BH UK; 5 Department of Oncology, University of Oxford, Oxford, OX1 4BH UK; 6 School of Medicine, University of California, San Diego, San Diego, CA 92093 USA

**Keywords:** Activin A, Digital Spatial profiling, Colorectal Cancer, Tumor microenvironment, Metastasis, Metastasis, Cancer, Gastrointestinal cancer, Colorectal cancer

## Abstract

Advanced colorectal cancer (CRC) continues to present with poor survival and treatment options remain limited. We have shown that increased activin A (activin) expression in the tumor microenvironment (TME) is associated with poor outcome in a cohort of stage III and IV CRC patients. Here, we hypothesized that activin promotes stage specific outcomes in CRC, enhancing metastasis and tolerance in late-stage CRC exclusively. We employed Digital Spatial Profiling (DSP) technology on a cohort of stage II and III CRC patient tissue samples obtained at the time of curative surgery to show that activin co-localization was associated with increased mitogenic signaling, proliferation, and immunosuppression in stage III, but not stage II, CRCs. Furthermore, we found strong linear correlations between markers of immunosuppression and signaling proteins in activin (+) areas, an effect that was not observed in activin (-) areas of tissue. Taken together these data suggest activin exerts pro-metastatic and immunosuppressive effects in stage III, but not stage II, CRC providing an attractive therapeutic target for advanced CRC.

**Supplementary Information:**

The online version contains supplementary material available at 10.1038/s41598-025-91853-9.

## Introduction

Colorectal cancer (CRC) remains the second leading cancer diagnosis and second highest mortality rate world-wide^1^. Despite improvements in screening rates and treatment options for advanced CRC, five-year overall survival rates are as low as 15% in stage IV patients, highlighting the clinical need for novel treatment options in late stage CRC^2^. Moreover, the number of CRC diagnoses in populations aged 20–35 years is on the rise in the US^3,4^ and young-onset CRCs tragically presents at later stages^5–9^. As screening for colorectal cancer is not recommended before age 45, we are experiencing a disproportionally growing number of advanced cancers in the younger population with a need for tailored therapeutics.

Metastasis, the multi-step invasive process that includes remodeling of the extracellular matrix (ECM) and evasion of the immune system^10^, is the primary cause of death for more than 90% of patients with cancer^11^. Targeting of each step, respectively, is an ongoing strategy. Immune checkpoint markers are expressed on immune cells (i.e. PD-1, CTLA-4, CD25) and on tumor cells (PD-L1) and together inhibit immune cell activation and elimination of tumor cells^12,13^. Recent work has led to the development of several immune checkpoint inhibitor therapies which can directly inhibit the PD-1/PD-L1 interaction to promote cytotoxic elimination of tumor cells^14^. However, the majority of CRC patients do not benefit from immune checkpoint inhibitor therapy given the immunosuppressive nature of the ECM, providing a significant clinical gap in treatment options for patients^15^. Therefore, identifying alternative molecules and signaling pathways which can inhibit immunosuppression is critical to advancing late stage therapeutics in CRC.

Activin A (activin) is a member of the transforming growth factor-β (TGF-β) superfamily which provides growth-suppressive effects in early stages of cancer, but promotes metastasis as the cancer develops^16,17^. Activin can stimulate several signaling pathways including the SMAD, PI3K, and MAPK pathways, among others, which may be responsible for the contextual changes in cellular responses to activin stimulation^18–21^. We recently provided evidence that activin was associated with increased expression of CTLA-4, CD25, and PI3K activation in the tumor microenvironment (TME) suggesting that this molecule may be promoting expression of these checkpoint markers through the PI3K pathway^22^. Furthermore, activin has been shown to suppress T-cell activation in several forms of cancer^22,23^. In addition to polarizing immune cells, activin has a well described role in tissue repair and wound healing through fibroblast activation, suggesting a potential role for this cytokine in fibroblast activation and ECM remodeling in cancer^24,25^. Recently published data suggest that cancer associated fibroblasts (CAFs) secrete significant quantities of activin which promote both migration of tumor cells and remodeling of the ECM via CAFs^26^. Furthermore, activin expression in the serum is significantly higher in stage IV CRC when compared to all other stages and anti-activin therapeutics are well tolerated in humans, providing an attractive therapeutic target for late-stage CRC^27–29^.

Here, we performed Digital Spatial Profiling (DSP) analysis on stage II/III CRC tumor biopsy samples from a phase II, randomized, controlled clinical trial (QUASAR II) to identify stage-specific cellular signatures to include immune cells and signaling patterns relative to activin co-localization in situ. Using this approach, we aimed to test the hypothesis that activin stimulates ECM remodeling, immunosuppression, and mitogenic signaling in CRC tissue samples in a stage-dependent manner with the goal of targeting activin signaling in advance CRCs.

## Results

### QUASAR II patient data information

The DSP analysis was performed on 30 patients from the QUASAR II clinical trial. The patients were matched across age (53–78 years old), sex (14 females, 16 males), and stage (16 stage III patients, 14 stage II patients) (Table 1). N stage and T stage information are also listed in Table 1. Mutation information for microsatellite-instability (MSI), Chromosomal Instability (CIN), Kras (codon 12, 13, 61 mutation), and the proto-oncogene Braf (V600E mutation) is also provided for all of the patients included in the DSP analysis (Table 1). Immunofluorescent images generated by the DSP staining protocol were independently scored by separate investigators and the average score was reported. No differences were observed in CD45 staining across stage II (1.08 ± 0.19 scoring units) and stage III (1.18 ± 0.15 scoring units) patients. The length of overall survival (days), length of recurrent free survival (days), and recurrence are also provided for the patients included in the DSP analysis (Table 1).

**Table 1:** DSP patient cohort. Information for age (years), sex, MSI status (0 = MSS/MSI-Low; 1 = MSI-High), CIN (0 = chromosomally stable, 1 = chromosomal instability), KRAS (0 = Wild-Type; 1 = codon 12, 13, 61 mutation), BRAF (0 = Wild-Type; 1 = V600E mutation), CD45 score (0 = no staining, 1 = intermediate staining, 2 = high staining), stage, recurrence (0 = no recurrence; 1 = recurrence), time to recurrence, and overall survival are provided.

Age	Sex	MSI	CIN	KRAS	BRAF	CD45 Score	Stage	T Stage	*N* Stage	Recurrence	Time Recurrent Free (Days)	Overall Survival (Days)
61	Female	0	1	0	0	2.0	3	0	1	0	1851	1851
63	Male	0	1	0	0	1.5	3	1	1	0	961	961
53	Female	0	1	0	0	1.3	3	0	1	0	1588	1588
75	Male	N/A	1	0	0	0.5	3	0	1	0	1834	1834
59	Female	1	1	N/A	N/A	1.3	3	0	2	1	707	1569
53	Male	0	1	N/A	N/A	2.0	3	0	1	0	1477	1477
62	Female	0	1	1	0	1.8	3	1	1	1	701	1841
54	Female	0	1	1	0	0.3	3	0	2	0	1826	1826
69	Female	0	1	0	1	1.2	3	0	0	0	1407	1407
65	Female	0	1	0	1	1.2	3	0	1	0	1324	1324
70	Female	0	0	0	0	0.5	3	0	1	0	1437	1437
68	Female	0		0	0	1.7	3	0	1	0	2489	2489
74	Male	0	1	0	1	1.0	3	1	2	1	112	256
69	Male	0	0	0	0	1.0	3	0	1	0	1420	1420
57	Male	0	0	0	0	0.0	3	0	0	0	1848	1848
64	Male	0	0	0	0	1.5	3	0	0	0	1504	1504
57	Female	0	1	1	0	2.0	2	0	0	0	1821	1821
54	Female	1	1	0	0	1.0	2	0	0	0	1911	1911
60	Female	0	1	N/A	N/A	1.0	2	1	1	0	2149	2149
62	Male	0	1	0	1	1.0	2	0	0	0	1905	1905
66	Male	0	1	1	N/A	1.5	2	0	0	0	2211	2211
64	Male	N/A	1	0	0	1.5	2	0	1	0	1503	1503
61	Male	0	1	1	0	2.0	2	1	0	0	1935	1935
57	Male	0	0	0	1	1.0	2	1	0	0	1899	1899
60	Female	0	1	1	0	0.8	2	1	0	0	1467	1467
75	Male	0	1	0	0	0.0	2	1	0	0	1163	1163
68	Female	0	1	N/A	1	0.0	2	1	0	0	689	689
72	Male	0	1	1	0	2.0	2	0	0	0	1456	1456
78	Male	0	1	0	0	0.0	2	1	1	1	1448	1489
69	Male	0	1	1	0	1.3	2	0	1	0	1050	1050

### CRCs display stage-specific and activin-dependent signaling patterns

**Fig. 1:**
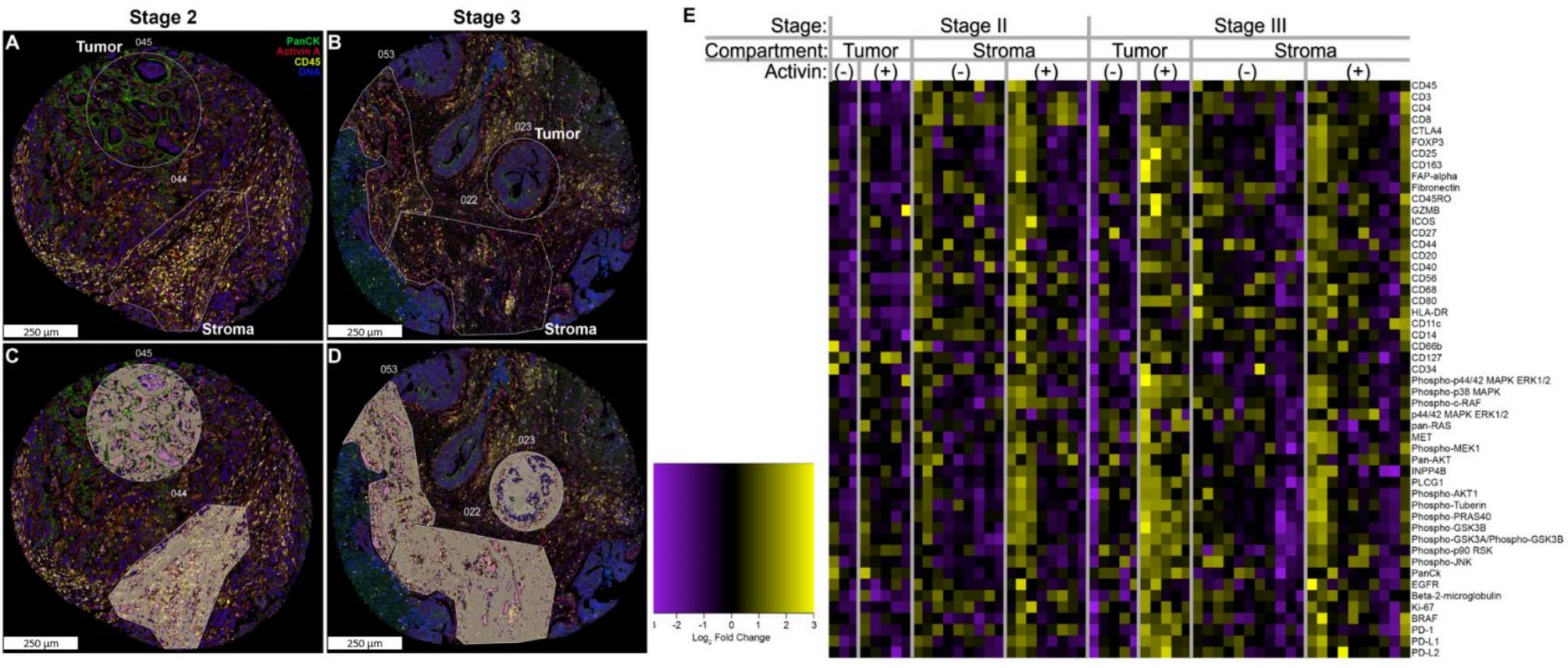
Signaling patterns in the TME are both activin- and stage-dependent. DSP technology was employed to separate regions of interest (ROIs) into stroma or tumor containing samples. (**A-B**) Representative ROIs labeled as tumor or stroma, (**C-D**) these images were further separated into activin positive (pink) and activin negative (gray) areas of illumination (AOIs) prior to collection and quantification. (**E**) Representative heatmap of the expression of each quantified protein within each sample when separated across stage, tissue compartment, and activin co-localization which identified the strongest heat sig-natures in the stage III tissue samples where activin was found.

Activin was first identified to stimulate the SMAD pathway through “canonical” signaling, however several studies have identified “non-canonical” pathways activated by activin including the PI3K and MAPK pathways^22,30–32^. To determine if non-canonical signaling was stage-dependent, we performed the DSP analysis on a TMA from the QUASAR II study which included both stage II and stage III CRC tissue samples^33^. The DSP method generated fluorescent images which were separated into tumor- or stroma-dominant ROIs (Fig. 1A-B). These images were further separated into activin (+) (pink) or activin (-) (grey) areas of illumination (AOIs) (Fig. 1C-D) which allowed for quantification of > 50 proteins relative to activin co-localization in situ. Separation of the tissue compartments across stage revealed that activin (+) AOIs contained the strongest heat signatures when compared to activin (-) AOIs (Fig. 1E), suggesting that the greatest increase in our proteins of interest occurred in regions of the tissue where activin was found. Furthermore, the effect of activin within the tumoral compartment appeared to be stage III exclusive. Changes in heat signature across activin (+) and (-) AOIs were not observed in stage II tumor samples, however there appeared to be an increase in heat signatures in several of the proteins included in our DSP panel in the activin (+) AOIs when compared to activin (-) AOIs in stage III tumor samples. These data suggest that activin exerts stage-specific effects within the tumoral compartment of the TME. For a list of all of the quantitative antibodies included in our DSP analysis see Supplemental Table 1.

### In stage III, but not stage II CRC, activin polarizes tumor cell signaling

**Fig. 2:**
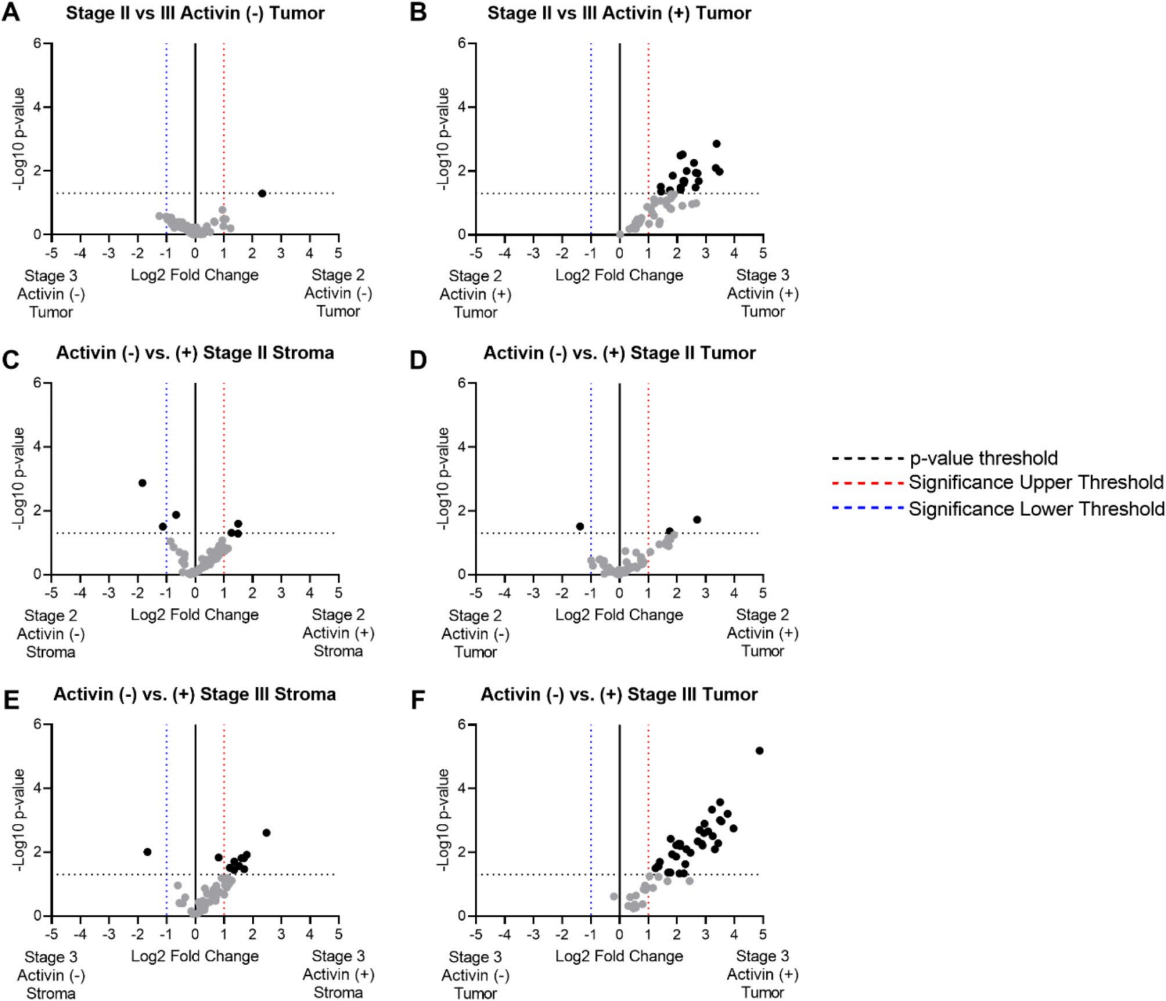
Activin exerts the greatest effect in stage III tumoral CRC tissue samples. Volcano plots were generated to determine where the most differentially expressed proteins could be identified. (**A**) Only one differentially expressed protein was observed across stage in activin (-) AOIs, (**B**) however several proteins were upregulated in stage III activin (+) AOIs compared to stage II. (**C-D**) Relatively few significant changes in protein expression were observed across activin (-) and (+) AOIs in stage II samples regardless of tissue compartment. (**E**) Activin (+) AOIs displayed an upregulation in several proteins compared to activin (-) AOIs in stage III stromal samples, and (**F**) the greatest number of significantly upregulated proteins were observed in stage III activin (+) tumoral samples which also displayed the greatest amount of significance.

To identify variables associated with the greatest number of significantly differentially expressed proteins, we generated several volcano plots to determine how stage, activin, or compartment each contributed to these changes. Only one differentially expressed protein was identified when comparing stage II and stage III tissue samples in the tumoral compartment of activin (-) AOIs (Fig. 2A). Interestingly, several proteins displayed a significant increase in expression in stage III when compared to stage II in the activin (+) AOIs of the tumoral compartment (Fig. 2B). These two pieces of data suggest stage-specific changes were localized to regions of CRC tissue where activin was found exclusively.

We further explored the potential of activin-dependent outcomes by comparing across activin co-localization within stage and tissue compartment. Relatively few changes in protein expression were observed when comparing activin (-) and activin (+) AOIs in the stage II samples regardless of tissue compartment (Fig. 2C-D). However, stage III tissue samples displayed significant changes in expression of several proteins across activin co-localization in the stromal compartment (Fig. 2E). The greatest number of significantly differentially expressed proteins were found when comparing the activin (+) and (-) AOIs in the tumoral compartment of stage III tissue samples. Moreover, each of these proteins were found to be upregulated in the activin (+) AOIs suggesting that activin may be responsible for stimulating the increase in protein production. These data suggest that activin-dependent changes in protein expression are stage III specific, further supporting the hypothesis that cellular responses to activin shift as CRC progresses.

### In stage III, but not stage II CRC, activin stimulates PI3K and MAPK activation, as well as Ki-67 and PD-1 expression

**Fig. 3:**
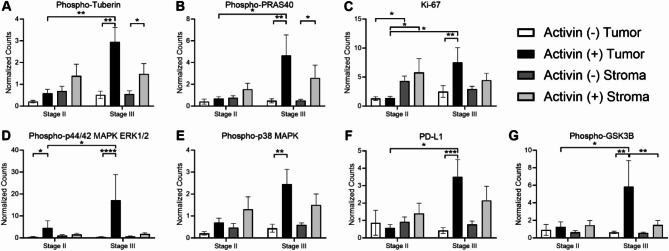
Activin co-localization is associated with mitogenic signaling, proliferation, and immune evasion. Activin co-localization in stage III CRC tissue samples was associated with increased levels of two PI3K signaling proteins (**A**) Phospho-Tuberin and (**B**) Phospho-PRAS40 regardless of tissue compartment. (**C**) Increases in the proliferative marker Ki-67 were observed in stage III activin (+) tumor sections and in the stroma of stage II tissue samples regardless of activin co-localization. (**D**) Increases in Phos-pho-p44-42 MAPK ERK1/2 were observed exclusively in activin (+) tumoral tissue sections, independent of stage. (**E-G**) Activin (+) AOIs in the tumoral compartment of stage III tissue sections displayed significant increases in Phospho-p38 MAPK, the immunosuppressive marker PD-L1, and Phospho-GSK3β. Data analyzed via linear mixed modeling with Benjamin-Hochberg correction test (*p < 0.05, **p < 0.01, ***, *p* < 0.001, ****p < 0.0001; *n* = 3, 5, 8, 9, 10, or 11).

We previously provided evidence that activin stimulates the PI3K pathway in human CRC tissue and enhances migration of CRC cells in vitro^22^. To further investigate the potential for this pathway in a stage-dependent context, we measured protein expression of several markers of the PI3K pathway in stage II and III CRC tissue samples relative to activin. We found that activin co-localization was associated with an increase in Phospho-Tuberin in both the tumoral (2.95 ± 0.67 normalized counts) and stromal (1.48 ± 0.47 normalized counts) compartments of stage III tissue samples when compared to activin (-) AOIs (tumor: 0.52 ± 0.17 normalized counts; stroma: 0.55 ± 0.14 normalized counts) (Fig. 3A). Similarly, Phospho-PRAS40 expression was increased in stage III CRC tissue samples in activin (+) AOIs of the tumoral (4.65 ± 1.89 normalized counts) and stromal (2.60 ± 1.17 normalized counts) compartments when compared to activin (-) AOIs (tumor: 0.48 ± 0.18 normalized counts; stroma: 0.52 ± 0.11 normalized counts) (Fig. 3B). However, no significant changes in expression of Phospho-Tuberin or Phospho-PRAS40 were observed across any conditions in stage II tissue samples (Fig. 3A-B). Additionally, activin co-localization was associated with an increase in Phospho-p44/42 MAPK ERK 1/2 in the tumoral compartment exclusively of stage II (4.48 ± 3.31 normalized counts) and stage III (17.17 ± 11.71 normalized counts) tissue samples when compared to activin (-) AOIs (stage II: 0.36 ± 0.18 normalized counts; stage III: 0.35 ± 0.12 normalized counts) (Fig. 3C). A significant increase in Phopsho-p38 MAPK was only observed in stage III tissue samples of the activin (+) tumoral AOIs (2.46 ± 0.66 normalized counts) when compared to activin (-) tumoral AOIs (0.44 ± 0.18 normalized counts) (Fig. 3D). These effects were coupled to increases in the proliferation marker Ki-67 and the immune evasion marker PD-L1 in stage III activin (+) tumoral AOIs (Ki-67: 7.54 ± 2.54 normalized counts; PD-L1: 3.52 ± 0.99 normalized counts) when compared to stage III activin (-) tumoral AOIs (Ki-67: 2.51 ± 1.00 normalized counts; PD-L1: 0.42 ± 0.17 normalized counts) (Fig. 3E-F). A significant increase in Phospho0GSK3B was also observed in activin (+) AOIs found in the tumoral compartment of stage III tissue samples (5.84 ± 2.98 normalized counts) when compared to activin (-) AOIs in the same compartment and stage (0.62 ± 0.15 normalized counts) (Fig. 3G).

### In stage III, but not stage II CRC, activin co-localizes with regulatory T-cells and is associated with increased markers of poor prognosis

**Fig. 4:**
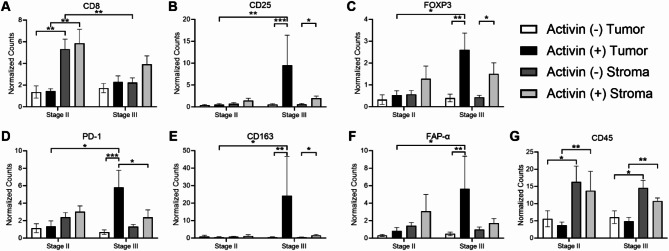
Activin co-localization is associated with immunosuppression in stage III exclusively. (**A**) Levels of the cytotoxic T-cell marker CD8 were significantly elevated in the stroma of stage II tissue samples which was not observed in stage III. (**B-D**) Activin (+) AOIs in stage III tissue samples displayed significant increases in the immunosuppressive markers (**B**) CD25, (**C**) FOXP3, and D) PD-1. Increased levels of the suppressive TAM marker (**E**) CD163 were also observed in stage III activin (+) AOIs in the tumoral compartment exclusively which was coupled to an increase in the fibroblast activation marker (**F**) FAP-α. (**G**) Increased CD45 expression was observed in the stromal compartment of the tissue sections regardless of activin co-localization or stage. Data analyzed via linear mixed modeling with Benjamin-Hochberg correction test (*p < 0.05, **p < 0.01, ***, *p* < 0.001, ****p < 0.0001; *n* = 3, 5, 8, 9, 10, or 11).

Published data by our group and others suggest that activin inhibits T-cell mediated tumor cell elimination in several forms of cancer^22,23,34,35^. Given the observed increase in circulating activin in late stage CRC^27^, we sought to determine if activin-associated T-cell suppression was stage specific in our cohort. The greatest expression of the cytotoxic T-cell marker CD8 was found in the stroma of stage II tissue samples regardless of activin co-localization ((-): 5.32 ± 0.93 normalized counts, *n* = 9; (+): 5.86 ± 1.29 normalized counts, *n* = 8) which was significantly higher than that of the tumoral compartment regardless of activin co-localization ((-): 1.36 ± 0.56 normalized counts, *n* = 3; (+): 1.46 ± 0.21 normalized counts, *n* = 5) (Fig. 4A). Interestingly, stage III activin (+) AOIs displayed the greatest expression of the T-cell marker CD3 (Supplemental Fig. 1A). In contrast, the greatest expression of both CD4 and CD8 were observed in stage II stromal samples (Fig. 4A and Supplemental Fig. 1B). The tumoral compartment of stage III activin (+) AOIs displayed the highest expression of regulatory T-cell markers CD25 (9.53 ± 6.87 normalized counts, *n* = 5) (Fig. 4B) and FOXP3 (2.61 ± 0.76 normalized counts, *n* = 5) (Fig. 4C). Activin (+) AOIs in the stromal compartment of stage III CRC samples also displayed significantly increased expression of CD25 (1.96 ± 0.51 normalized counts, *n* = 10) and FOXP3 (1.50 ± 0.51 normalized counts, *n* = 10) (Fig. 4C) when compared to activin (-) AOIs in the same compartment (CD25: 0.61 ± 0.16 normalized counts, *n* = 11 ; FOXP3: 0.43 ± 0.10 normalized counts, *n* = 11). Additionally, stage III activin (+) AOIs in the tumoral compartment were found to have the greatest expression of several markers of poor prognosis for CRC patients including CD163 (24.23 ± 22.39 normalized counts, *n* = 5) (Fig. 4D), FAP-α (1.95 ± 0.59 normalized counts, *n* = 5) (Fig. 4E), and PD-1 (5.79 ± 1.96 normalized counts, *n* = 5) which was significantly higher than activin (-) AOIs in the same compartment of stage III patient samples (CD163: 0.47 ± 0.019 normalized counts; FAP-α: 0.49 ± 0.20 normalized counts; PD-1: 0.68 ± 0.25 normalized counts, *n* = 5). Activin (+) AOIs in the stroma also displayed significantly increased expression of CD163 (1.56 ± 0.53 normalized counts, *n* = 10) FAP-α (1.72 ± 0.52 normalized counts, *n* = 10 ), and PD-1 (2.37 ± 0.86 normalized counts, *n* = 10) when compared with stromal activin (-) AOIs from stage III patients (CD163: 0.44 ± 0.10 normalized counts; FAP-α: 0.98 ± 0.29 normalized counts; PD-1: 1.31 ± 0.25 normalized counts, *n* = 11) (Fig. 4E-G).

### TCGA data set confirms increased activin expression is correlated with increased markers of poor prognosis in CRC tissue samples

**Fig. 5:**
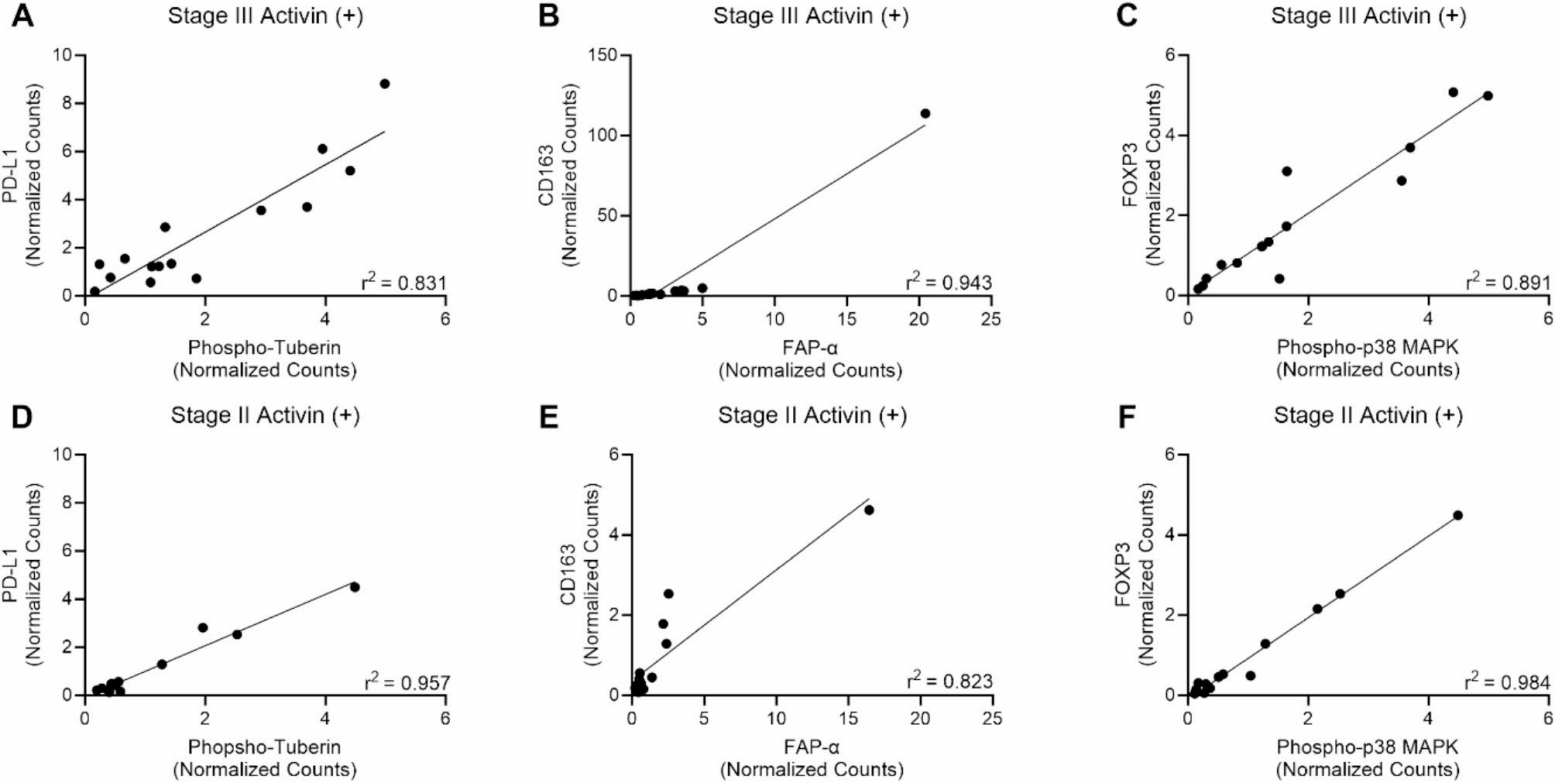
Activin (+) AOIs display several correlations not observed in activin (-) AOIs. Activin co-localization was associated with the correlation of (**A**) PD-L1 and Phospho-Tuberin, (**B**) CD163 and FAP-α, and (**C**) FOXP3 and Phsopho-p38 MAPK in stage III tissue samples regardless of tissue compartment. (**D-F**) Similar correlations were observed in stage II activin (+) AOIs. These effects were not observed in activin (-) AOIs (see supplemental Fig. 2). Data analyzed via linear correlation with R-squared values reported to represent the goodness of fit.

In melanoma, elevated levels of *INHBA* mRNA (the mRNA that encodes activin) correlate with resistance to anti-PD-1 therapy^23^ suggesting activin plays a critical role in mediating the PD-1/PD-L1 axis. We investigated potential pathway correlations with PD-L1 in activin (+) AOIs and found that PD-L1 correlates with Phospho-Tuberin in activin (+) AOIs exclusively (Fig. 5A and D, and Supplemental Fig. 2A and 2D) suggesting that activin may stimulate PD-L1 expression through activation of the PI3K pathway. Additionally, activin stimulates ECM remodeling to facilitate metastasis through increased expression of fibroblast activation protein-α (FAP-α) on CAFs and CD163 on tumor associated macrophages (TAMs)^26,36,37^. Therefore, we included CD163 and FAP-α in our linear correlation analysis and found strong correlations in activin (+) AOIs (Fig. 5B and E) which were not observed in activin (-) AOIs (see Supplemental Fig. 2B and 2E). We also investigated the potential for the MAPK pathway in suppressing T-cells and found that FOXP3/Phospho-p38 MAPK displayed significant correlations which were exclusive to activin (+) AOIs (Fig. 5C and F). The correlation of expression between these proteins is lost in the activin (-) AOIs of the tissue regardless of stage (see Supplemental Fig. 2C and 2 F). Please see Supplemental Table 2 for statistical information for all DSP data.

**Fig. 6:**
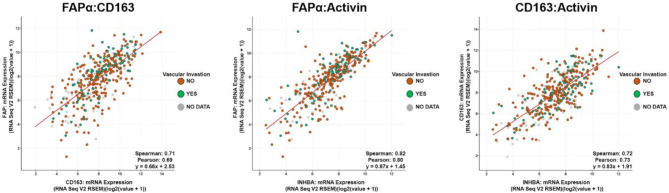
Genomic database correlation analysis confirms protein expression association data between Activin A (INHBA), CD163, and FAP alpha. mRNA expression analysis shows positive correlations between (**A**) INHBA and CD163, (**B**) INHBA and FAP alpha, (**C**) and CD163 and FAP alpha. Green dots represent data points in which the patient exhibited vascular invasion, orange indicates absence of vascular invasion, and gray indicates no data acquired on vascular integrity. Linear regression analysis was run on cBioPortal for Cancer Genomics and tested Spearman and Pearson Correlation. Data sets acquired from The Cancer Genome Atlas Firehose Legacy.

We then searched The Cancer Genome Atlas Firehose Legacy data set to perform multiple correlation analyses. We found a positive correlation between the mRNA for FAPα and CD163 when performing a Spearman correlation analysis and a Pearson correlation analysis (Fig. 6A). A similar positive correlation was found between the mRNA for activin (*INHBA*) and FAPα, and between INHBA and CD163 when the same correlation analyses were performed (Fig. 6B **& C**). Taken together, these data suggest activin co-localization in the TME promotes CD163 and FAPα expression to facilitate a tumor- tolerant environment.

## Discussion

Currently, surgical resection of the tumor with curative intent is the most common initial treatment option for both stage II and III CRC patients. Many high-risk stage II CRC patients receive adjuvant chemotherapy of fluorouracil and folinic acid treatment which has shown to improve survival by ~ 4%^38^. Treatment recommendations for stage II CRC patients varies based upon the presence of high-risk features of recurrence^39^, while 12 cycles of oxapliplatin and fluoropyrimidine combination therapy is the standard of care for stage III patients following surgery^40^. Given the lack of changes in protein expression observed in the stage II tissue samples across activin (+) and (-) AOIs in our cohort and the fact that circulating levels of activin do not differ between stages II and III^29^, activin may not be an optimal biomarker of disease or therapeutic target early in CRC. However, the TME becomes significantly more pro-metastatic and tumor tolerant in stage III tissue samples in activin positive tissue suggesting a potential shift in cellular responses to activin as disease progresses. Activin and TGF-β are both capable of activating the SMAD pathway^41,42^, therefore TGF-β was included in our previously published work where we reported that high ratios of activin: TGF-β were associated with improved overall survival within this QUASAR II cohort (stages II and III exclusively) when quantified via IHC^22^, while the opposite was observed in a smaller, separate cohort which was comprised of mostly stage III or stage IV tissue samples^43^. These distinct changes in response to activin highlight the need to generate and publish spatial proteomic data of the TME of CRC tissue samples to identify markers and targets for aggressive disease.

Interestingly, some of the effects of activin appear to also be cancer type-specific. In lung cancer, high levels of activin are a prognostic factor of survival independent of stage, an effect not observed in CRC^44^. The data presented here and in our recently published work suggests that activin exerts immunosuppressive effects on T-cells in CRC^22^. A similar effect is observed in melanoma^23^ and breast cancer^34^ suggesting activin may be an effective target for immunotherapies in several forms of cancer. However, in the setting of lung cancer, activin promotes CD4 to CD8 T-cell communication to enhance cytotoxicity and tumor cell elimination^45^. Many of these opposing effects are likely attributed to the complex nature of the activin signaling system which includes several subtypes of receptors which have varying expression levels that are both context-dependent and cell-specific^19,45^.

Similar to what others have reported in the context of cancer^46^, we observed significant immune cell polarization in the presence of activin. Recently published data identified that CAFs and TAMs found at the peritumoral border in melanoma communicate to regulate CD8 T-cell trafficking into the tumor and that activin-associated reprogramming of both CAFs and TAMs promoted T-cell exclusion from the TME, immunosuppression of several immune cells, and ECM remodeling to promote metastasis^36^. More specifically, the published work identified that activin-associated changes of CAFs and TAMs included high expression of CD163 on TAMs and FAP-α on CAFs leading to CD8 T-cell exclusion and immunosuppression^36^. Our observed correlations of FAP-α and CD163 in activin (+) AOIs exclusively further support the hypothesis that activin drives immunosuppression and ECM remodeling in the TME. Interestingly, this correlation was not stage-specific providing an activin-mediated signaling pattern vital to ECM remodeling and immunosuppression that may be consistent throughout disease progression.

We did not observe a significant increase in phospho-AKT1 in our DSP analysis, nor did we observe any increase in pan-AKT despite seeing increases in Phospho-Tuberin and Phospho-PRAS40. However, an increase in phospho-GSK3B was observed in activin (+) AOIs which has been shown to cross-talk with Phospho-Tuberin via Beta-catenin signaling^47^. Additionally, activin A stimulates mTOR activity which can then stimulate phosphorylation of PRAS40^48,49^. Therefore, it is possible that activin is stimulating phosphorylation of PRAS40 and Tuberin via Akt-independent mechanisms.

Several of the highly effective treatment options for CRC patients are designed for patients with specific mutations (i.e. KRAS, EGFR, VEGF) or specifically target the immune system, however these approaches are not effective in the majority of CRC patients^15^. The data provided here suggests that tumoral activin is associated with increased expression of proliferation, immunosuppression, and CAF activation suggesting that targeting activin may be effective in multiple forms of CRC regardless of mutations and MSI/MSS status. Serum levels of activin are elevated in patients with malignant pleural mesothelioma and elevated levels are also associated with cancer cachexia when compared to non-small cell lung cancer or benign lung lesions^50^. Taken together with our data which suggests that activin exerts stage-specific effects to promote aggressive CRC, activin may provide an attractive biomarker for identifying patients with severe disease. Furthermore, our immune-related data suggest that there may be potential to use activin as an indicator of how effective immunotherapy will be in CRC patients. Activin inhibition in humans is well-tolerated providing an attractive therapeutic target for a potentially high-yield clinical trial in CRC^28^.

## Conclusions

These data provide evidence that (i) stage-specific effects in CRC tissue are activin-dependent, (ii) activin stimulates the PI3K pathway to promote tumor cell proliferation and expression of immunosuppressive markers, (iii) activin activates the MAPK pathway to support immunosuppression, and (iv) mediates macrophage and fibroblast interactions to facilitate metastasis. Together, these data suggest activin’s pro-metastatic and immunosuppressive effects are stage-specific providing an attractive target for late-stage CRC therapeutics.

## Materials and methods

### Patient cohort tissue microarray

The cohort employed included patients from the QUASAR 2 study which was a phase 3, randomized, controlled trial performed as previously described^33^. Good Clinical Practice, European Directives 2001/20/EC and 2005/28/EC, and the Declaration of Helsinki. Study approval was obtained from the West Midlands Research Ethics Committee (Edgbaston, Birmingham, UK; REC reference: 04/MRE/11/18) prior to completion of the study. Recruitment, tissue collection, and storage were all performed as previously described^22,33,51^. One slide including a tissue microarray (TMA) of 116 patient samples was employed for DSP analysis (see below).

### Digital Spatial profiling (DSP, NanoString)

The DSP assay (NanoString Technologies, Seattle, WA, USA) which includes the staining, region of interest selection, area of illumination selection, normalization, and analysis were performed as previously described^22,52^. Deparaffinization followed by antigen retrieval was performed on the formalin-fixed, paraffin embedded tissue sections. Slides were then stained with 56 antibodies which are conjugated through a photocleavable linker with quantifiable oligonucleotide “barcodes” (see Supplemental Table 1). Simultaneously, the slide was stained with three morphology markers (fluorescent antibodies): CD45, PanCK, and DNA (SYTO13, all NanoString Technologies). Additionally, AF647 was conjugated to activin using the Alexa Fluor 647 Antibody Labeling Kit (ThermoFisher Scientific, Waltham, MA, USA) which was also used as a morphology marker. This generated an anti-activin antibody conjugated with the AF647 fluorophore at 10 µM which was further diluted to 0.25 µg/mL. All fluorescent detection antibodies were stained at a 1:25 dilution from the stock concentration provided by NanoString. Similarly, the quantitative morphology antibodies were stained at a 1:40 dilution from the stock concentration provided by NanoString. All dilutions were performed at the recommended concentrations that are listed in the DSP protocol provided by NanoString.

Following fixation, fluorescent images were taken using the fluorescent microscope built into the DSP machine and regions of interest (ROIs) were selected in areas where dense expression of activin, CD45, and/or PanCK were observed. These ROIs were further separated in activin (+) and activin (-) areas of illumination (AOIs). Activin (+) AOIs were first exposed to ultra-violet (UV) light to permit cleavage of the quantifiable barcodes and barcodes were subsequently collected into a 96-well plate. The process was then repeated on the activin (-) AOIs. Collected barcodes were quantified using an nCounter Sprint Profiler (NanoString Technologies) as previously described^22,53^. Data QC was performed to remove low quality samples and data points as previously described^22^. Samples with low nuclei count, high positive-control spike-in expression, low binding density, low surface area, and low field-of-view detection were removed. Negative controls were also included to permit background correction, and normalization was performed using the housekeeping proteins GAPDH and S6. Following data pruning via QC, a total of 30 unique patient samples were included in the DSP analysis.

### Statistical analysis

Analysis of DSP data was performed via Linear mixed modeling (LMM) with Benjamin-Hochberg correction test (NanoString DSP Analysis Suite Software) as previously described^22^ (*p < 0.05, **p < 0.01, ***p < 0.001, ****p < 0.0001). The heatmap included in Fig. 1 was generated using Rstudio 2022.07.02 and the package “gplots”.

## Electronic supplementary material

Below is the link to the electronic supplementary material.

Supplementary Material 1

## Data Availability

All relevant data are included in the manuscript or in the Supplementary Materials.
