# Supplementary material for: Activin A affects colorectal cancer progression and immunomodulation in a stage dependent manner
Source: Sci Rep. 2025 Mar 12;15:8509. doi: 10.1038/s41598-025-91853-9 (PMC11903883; doi:10.1038/s41598-025-91853-9)
Supplement: Supplementary file 1 — Supplementary Material 1 [file 41598_2025_91853_MOESM1_ESM.docx]

**Supplemental Table 1:**
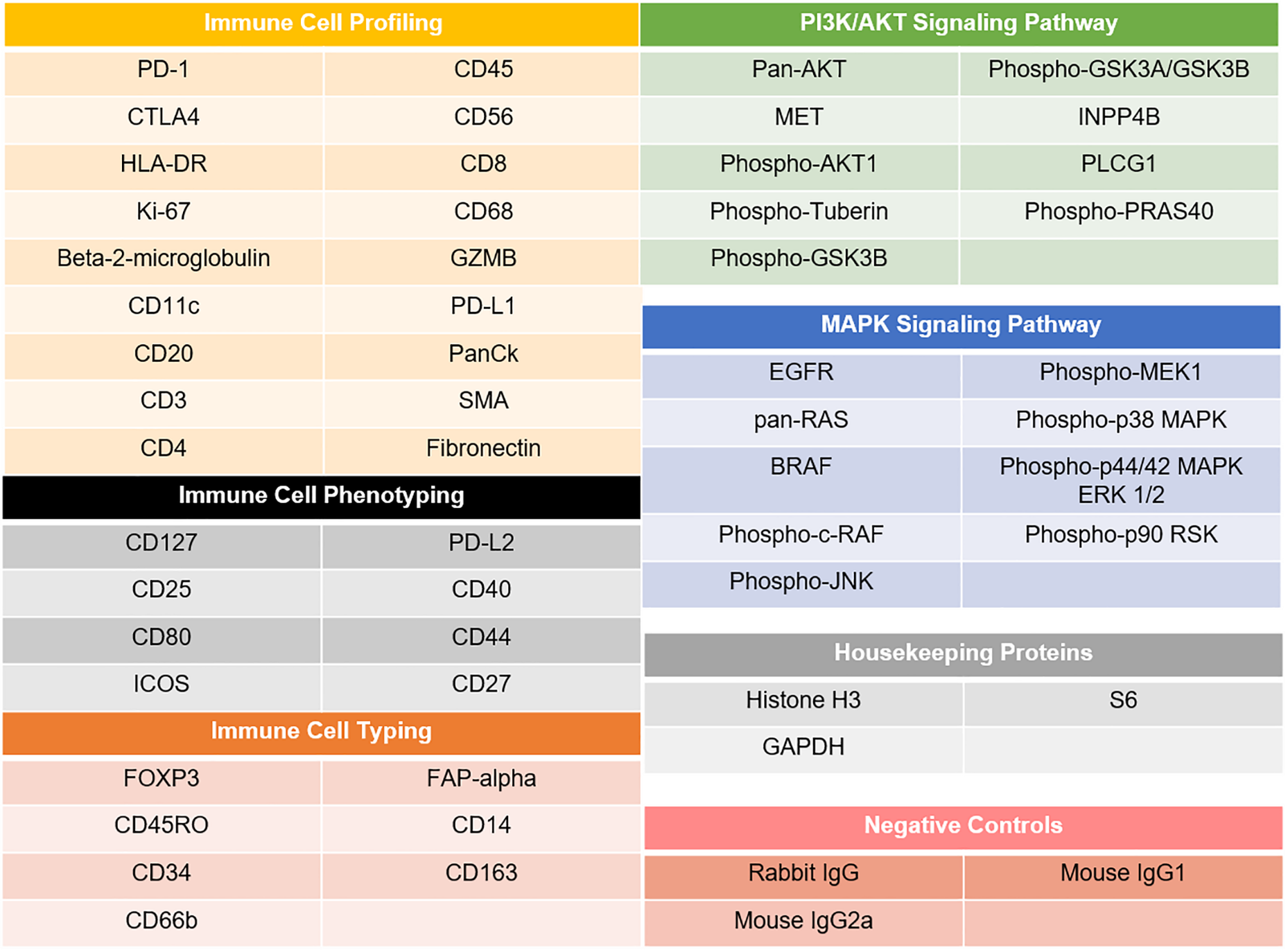
List of the quantitative markers included in the DSP analysis.

| Protein of Interest | Stage 2 Tumor Activin (-) n = 3 | Stage 2 Tumor Activin (+) n = 5 | Stage 2 Stroma Activin (-) n = 9 | Stage 2 Stroma Activin (+) n = 8 | Stage 3 Tumor Activin (-) n = 5 | Stage 3 Tumor Activin (+) n = 5 | Stage 3 Stroma Activin (-) n = 11 | Stage 3 Stroma Activin (+) n = 10 |
| --- | --- | --- | --- | --- | --- | --- | --- | --- |
| P-Tuberin | 0.21 ± 0.06 | 0.59 ± 0.18 | 0.69 ± 0.22 | 1.39 ± 0.54 | 0.52 ± 0.17 | 2.95 ± 0.67 | 0.55 ± 0.14 | 1.48 ± 0.47 |
| P-PRAS40 | 0.41 ± 0.23 | 0.68 ± 0.19 | 0.75 ± 0.20 | 1.55 ± 0.54 | 0.48 ± 0.18 | 4.65 ± 1.89 | 0.52 ± 0.11 | 2.60 ± 1.17 |
| Ki-67 | 1.31 ± 0.32 | 1.35 ± 0.29 | 4.32 ± 0.86 | 5.80 ± 2.35 | 2.51 ± 1.00 | 7.54 ± 2.54 | 2.94 ± 0.47 | 4.44 ± 1.20 |
| P-p44/42 MAPK ERK 1/2 | 0.36 ± 0.18 | 4.48 ± 3.31 | 1.03 ± 0.41 | 1.59 ± 0.44 | 0.35 ± 1.12 | 17.17 ± 11.71 | 0.67 ± 0.17 | 1.73 ± 0.57 |
| P-p38 MAPK | 0.21 ± 0.08 | 0.70 ± 0.20 | 0.47 ± 0.19 | 1.31 ± 0.57 | 0.44 ± 0.18 | 2.46 ± 0.66 | 0.59 ± 0.10 | 1.50 ± 0.50 |
| PD-L1 | 0.87 ± 0.72 | 0.57 ± 0.19 | 0.92 ± 0.29 | 1.40 ± 0.59 | 0.42 ± 0.17 | 3.52 ± 0.99 | 0.78 ± 0.19 | 2.15 ± 0.81 |
| CD8 | 1.36 ± 0.56 | 1.46 ± 0.21 | 5.32 ± 0.93 | 5.86 ± 1.29 | 1.72 ± 0.42 | 2.30 ± 0.55 | 2.24 ± 0.42 | 3.92 ± 0.79 |
| CD25 | 0.42 ± 0.16 | 0.54 ± 0.20 | 0.69 ± 0.28 | 1.45 ± 0.54 | 0.55 ± 0.19 | 9.53 ± 6.87 | 0.61 ± 0.16 | 1.96 ± 0.51 |
| FOXP3 | 0.33 ± 0.21 | 0.53 ± 0.21 | 0.57 ± 0.18 | 1.29 ± 0.57 | 0.40 ± 0.18 | 2.61 ± 0.76 | 0.43 ± 0.10 | 1.50 ± 0.51 |
| CD163 | 0.87 ± 0.76 | 0.52 ± 0.21 | 0.80 ± 0.30 | 1.26 ± 0.58 | 0.47 ± 0.19 | 24.23 ± 22.39 | 0.44 ± 0.10 | 1.56 ± 0.53 |
| FAP-α | 0.33 ± 0.11 | 0.83 ± 0.39 | 1.41 ± 0.39 | 3.07 ± 1.93 | 0.49 ± 0.20 | 1.95 ± 0.59 | 0.98 ± 0.29 | 1.72 ± 0.52 |
| PD-1 | 1.14 ± 0.51 | 1.35 ± 0.61 | 2.41 ± 0.51 | 3.02 ± 0.66 | 0.68 ± 0.25 | 5.79 ± 1.96 | 1.31 ± 0.25 | 2.37 ± 0.86 |

**Supplemental Table** **2:** Summary of the statistical information for each significantly differentially expressed protein.


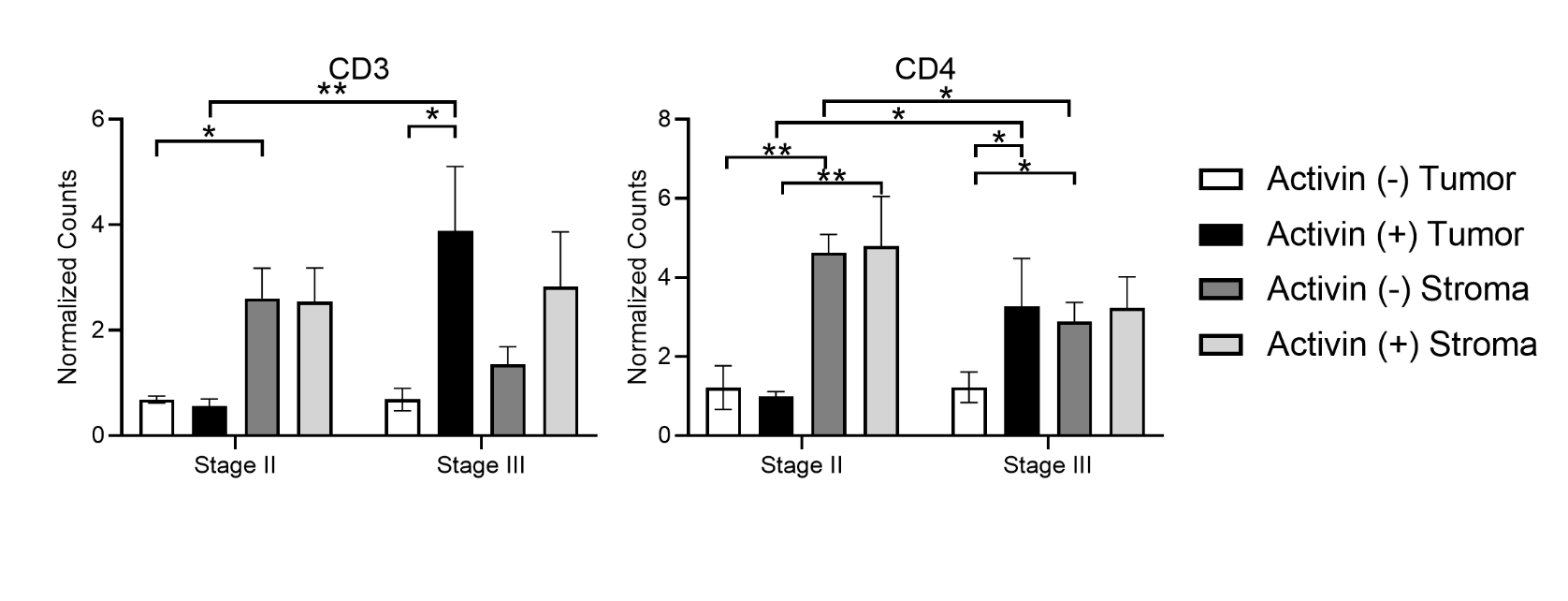
**Supplemental Figure 1:** Expression levels of CD3 and CD4 in stage II and III CRC tissue.


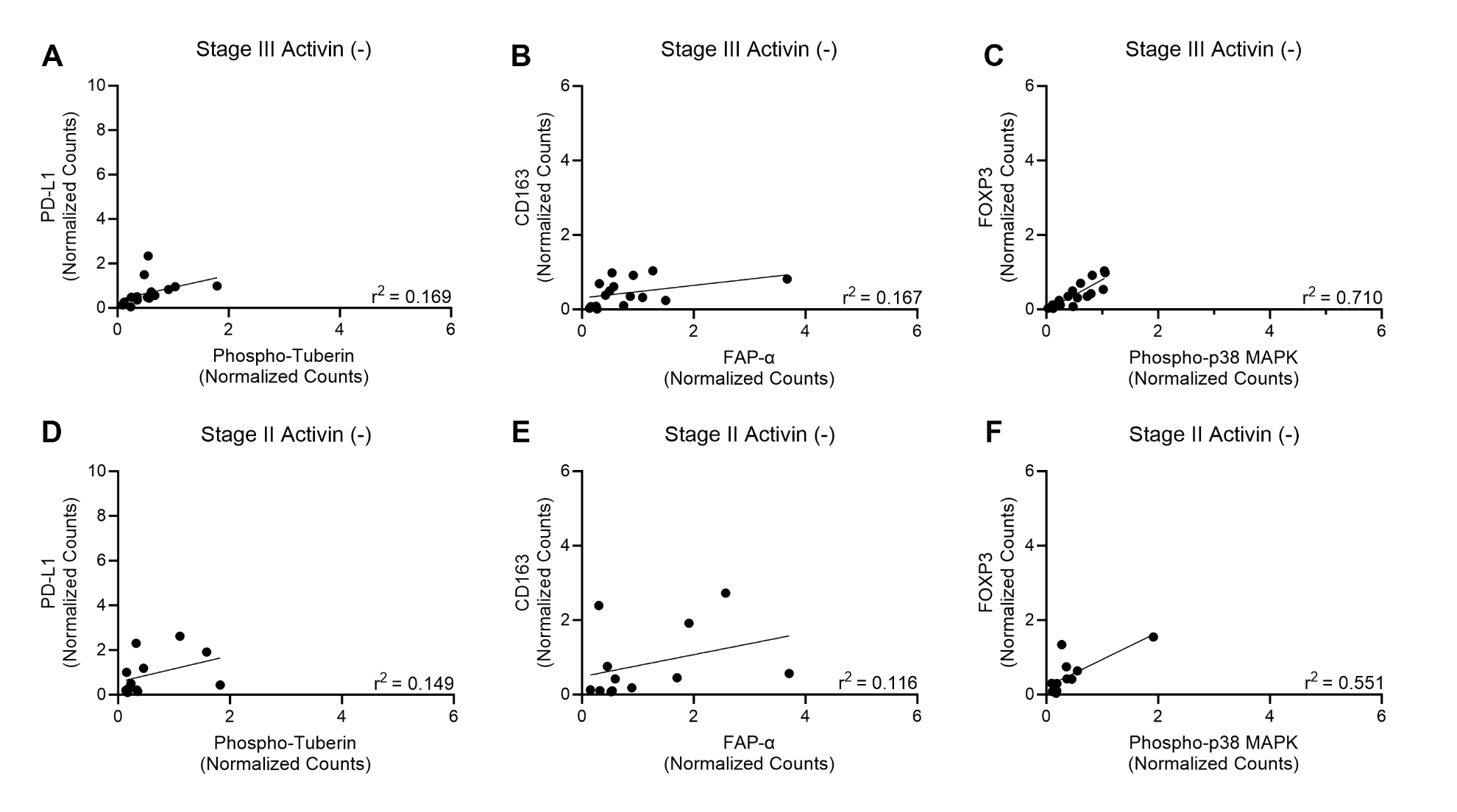
**Supplemental Figure 2**: Correlation plots in activin (-) AOIs.
